# Supplementary material for: Are carbon emissions trading and green financial instruments synergistic? -Comprehensive quantitative research based on content analysis
Source: PLoS One. 2024 Mar 7;19(3):e0298601. doi: 10.1371/journal.pone.0298601 (PMC10919601; doi:10.1371/journal.pone.0298601)
Supplement: S1 Data — (ZIP) [file pone.0298601.s001.zip › Data/地级市Z_F工作报告.zip!/╔╜╢1⁄2/▒⌡╓▌╩╨/└┤╫╘╣1⁄2╓┌║┼í╛┬φ┐╦╩2╛▌═°í┐_2013.docx]

各位代表:

现在，我代表市人民政府向大会作工作报告，请予审议，并请政协委员和其他列席人员提出意见。

一、2012年工作回顾

2012年，我们坚持以科学发展观为指导，牢牢把握主题主线，加快转变经济发展方式，促进经济持续健康发展，较好地完成了各项目标任务，实现了本届政府稳中有进的良好开局。

—综合实力大幅提升。预计地区生产总值、固定资产投资分别实现2015亿元、1230亿元，增长10.8%、22%。地方财政收入实现150.46亿元, 增长15.1%。金融机构本外币存款余额1697.7亿元，增长29.6%，增幅列全省第1位；贷款余额1495.1亿元，增长19%，增幅列全省第4位。预计城镇居民人均可支配收入实现25990元，增长15.3%，农民人均纯收入实现10143元，增长16%，首次突破“万元大关”。荣获全省县域经济跨越发展第一位。

—基础设施日趋完善。滨州港2个3万吨级码头、28公里深水岸线全面竣工。滨德高速按期通车，济滨高速开工奠基，德大铁路加快建设，滨港铁路一期健康运营、二期准备工作全面推进。投资15.1亿元，顺利完成12.5万亩的未利用地开发项目；启动实施了总投资67.2亿元、可带来60万亩未利用地效益的徒骇河雨洪水资源综合利用工程。整合港口、未利用地等各类资产200亿元，融资能力明显 增强。

—产业转型步伐加快。新增规模以上企业105家，预计规模以上工业增加值增长13%；新增省级以上科研载体59家，新增高新技术企业15家，高新技术产业产值实现1200亿元，增长23%以上；新增中国驰名商标3件、山东省著名商标20件、山东名牌产品16件；魏桥集团跻身世界500强，销售收入突破1800亿元；在沿海经济带上，相继开工投产总投资10亿元以上的项目9个、总投资100亿元以上的项目6个。黄河三角洲（滨州）国家农业科技园区、沿黄高效生态农业示范带建设扎实推进；71家示范园区的规模、质量、效益不断提升；农产品安全追溯管理控制中心全面建成；新增规模以上农业龙头企业5家、专业合作社28个；粮食生产实现“十连增”，预计达到311万吨；循环农业、机采棉等经验在全国推广。积极发展电子商务、冷链物流、网上配送等现代服务业；新引进兴业、招商、交通3家银行；成功举办全省春季糖酒商品交易会、第十届国际家纺文化节、黄河三角洲汽车展等大型节会；预计服务业实现增加值760亿元，增长10%。

—城乡面貌变化巨大。北环河绿化带建设、黄河二路水系景观绿化全部完成；秦皇河治理等工程成效显著；空气质量列全省13个二类城市第2位；市城区7个城中村完成整体搬迁；顺利通过“国家卫生城市”复审。扎实开展“林水会战”，新增造林22.7万亩，新建标准化方田3.9万亩，恢复新增蓄水能力4802万方；新建万亩林场6处、新规划生态湿地8处；建设市级生态文明村296个；4231个村居实现城乡环卫一体化。高度重视节能减排，预计万元GDP能耗同比下降3.7%以上；大力发展循环经济，14家企业、43个乡镇分别被命名为国家或省级环境友好企业、环境优美乡镇。

—改革开放不断深化。财税、科技、社会保障、医疗卫生等改革稳步推进。国家基本药物制度实现基层全覆盖,二级以下公立医院全部实行“先诊疗、后付费”，城乡基本医疗卫生制度初步建立；获得国家基本公共卫生服务项目考核第二名。继续推进公共资源交易制度改革，规范土地出让、建设工程招投标、政府采购、产权交易行为，全年市级交易额60.1亿元，节支增收3.4亿元；进一步转变政府职能，强化依法行政，加大政务公开，获得首届“中国阳光政府奖”。积极参与韩国丽水世博会山东活动周、鲁港经贸洽谈会，一大批项目达成合作协议；与世界77个国家和地区建立了良好的经贸关系，预计进出口总值62亿美元、实际利用外资5.3亿美元；全市上市公司达15家，全年在资本市场直接融资84亿元，其中首发上市融资列全省第1位。

—民生保障全面加强。市级财政用于社会民生投入143.8亿元，占财政支出的63.5%，比上年增长18.2%。农村低保标准由年人均1610元提高到“东部标准”的2300元，城市低保标准提高到每月人均400元。调整延伸公交线路15条，新增新能源公交车100辆；市城区供热面积由730万平方米增加到1000万平方米；开工建设保障性住房9755套，竣工完成率120.9%；完成农村住房建设、危房改造25486户；新增城镇就业8万人，农村劳动力转移就业13.9万人；去年承诺的16项民生实事全部兑现，人民群众衣食住行条件明显改善。

—社会事业稳步推进。教育事业迅速发展，素质教育全面实施，教育质量不断提高；公共文化服务体系日趋完善，文化创作更加繁荣，吕剧《杨广和》在第四届全国少数民族文艺会演中夺得9项大奖，文化产业迅速发展；竞技体育实现奥运会奖牌零的突破，群众体育广泛开展；人口计生指标全面完成；创建全国双拥模范城实现“两连冠”。深入推进社会管理创新，高度重视安全生产和食品安全，严厉打击违法犯罪，社会大局保持和谐稳定。其他各项事业得到全面发展。

在总结成绩的同时，我们也清醒地看到，工作中还存在许多困难和问题：一是发展中不平衡、不协调、不可持续的问题依然突出，自主创新能力不强，产业结构不合理，基础设施依然薄弱，经济增长的质量和效益不高。二是制约发展的体制机制障碍较多，行政许可和审批事项仍然过多，有的部门权责脱节、有权无责、职责不清、办事效率低。三是有些干部科学发展的能力不强，思想不够解放，缺乏干事创业的激情和士气，等等。这些问题，我们必须高度重视并认真加以解决。

经历了金融危机的磨炼，面对复杂严峻的国内外形势，我们深深地认识到，成功缘于实干，失败始于空谈。正处于发展攻坚期、转型关键期的滨州，面临的挑战前所未有。有信心才会有力量，有信心迈出的步伐才会更稳健。只要我们适应时代需要，顺应群众期盼，不为任何风险所惧，不为任何干扰所惑，勇于攻坚克难，敢于迎难而上，就一定能够打开滨州发展的新局面，就一定能够开创滨州发展的新境界！

在这里，我代表市人民政府，向勤劳智慧的全市人民，向大力支持我们工作的人大代表、政协委员和各民主党派、工商联、各人民团体、无党派人士、驻滨部队、武警官兵、公安干警，国家部委和省直机关及其驻滨单位、兄弟市和社会各界，向所有关心、支持、参与滨州改革发展的海内外朋友，表示衷心的感谢并致以崇高的敬意!

二、2013年目标要求

今年是落实党的十八大精神的开局之年，是实施“十二五”规划承前启后的关键之年。既要看到面临的困难和挑战，更要看到面临的机遇和有利条件，从最坏处准备，向最好处努力，牢牢把握工作主动权。

总体要求是：准确把握稳中求进的总基调，以提高经济增长质量和效益为中心，以生态发展为导向，以改善民生为根本，以产业提升为重点，以创新驱动为支撑，以廉政建设为保障，扎实开展“生态建设年”、“提质增效年”、“作风改进年”活动，促进新型工业化、信息化、城镇化、农业现代化同步发展，确保到2016年实现六个翻番、全面建成小康社会、再造一个新滨州的宏伟目标。

预期目标是：地区生产总值增长11.5%，地方财政收入增长13%，固定资产投资增长21%，规模以上工业增加值增长13%，进出口总额增长10%，社会消费品零售总额增长15%，城镇居民人均可支配收入增长12.5%，农民人均纯收入增长13%，人口自然增长率控制在5.6‰以内。万元地区生产总值能耗及主要污染物排放，全部完成省里下达的目标任务。

实现上述目标，必须认真总结经验，科学判断形势，在以下原则要求上形成共识。

—更加注重质量效益，坚持内涵发展。发展是解决所有问题的关键。我们既讲加快发展，又讲加快转调；既重视发展速度，更重视发展的质量、效益和可持续。在发展中调整，在调整中加速，把质量和效益体现在增强长期发展的后劲上，体现在提高人民群众的生活水平上。

—更加注重资源环境，坚持生态发展。生态文明建设不仅是一个发展问题，也是一个重大民生问题。我们既要注重经济发展，更要注重环境保护，必须进一步提高认识，推进“绿色、循环、低碳”发展，努力形成节约环保的产业结构、生产方式、生活方式。这是转变经济发展方式的重点任务和重要内涵。

—更加注重科技人才，坚持创新发展。科技是第一生产力，人才是第一资源。实现可持续发展必须依靠科技创新，人才是科技创新的决定性因素。未来区域发展的竞争力，取决于科技人才队伍的数量与质量，取决于自主创新能力的高低。这是转方式调结构的战略支撑。

—更加注重城乡统筹，坚持协调发展。做好城乡统筹是缩小城乡差距、地区差距，增强农村发展活力，促进城乡共同繁荣的迫切需要。城镇化是促进城乡统筹的重要载体，是实现产业结构、就业方式、人居环境等由“乡”到“城”转变的必然路径，是经济增长的巨大引擎。

—更加注重改善民生，坚持民本发展。社会保障是民生之安，关系到每一个人、每一个家庭的幸福。我们发展的目的，归根结底都是为了让人民群众过上更美好的幸福生活，真正享受到改革发展和现代化建设的成果，进一步促进社会公平正义。

到2016年全面建成小康社会，标志着滨州的现代化建设步入新阶段。实现这个目标，还需要我们付出长期艰苦的努力，要求我们紧密结合实际，把握好五个“更加注重”、坚持“五个发展”。只有这样，我们的经济发展才能又好又快，质量效益才能实实在在，才能更好地保障改善民生。越是在这样的关键阶段，越要立足实际、脚踏实地，在全市人民的共同努力下，在我们每个人的奋斗中把蓝图变为现实！

三、2013年工作重点

实现科学发展、追赶超越，必须切实增强紧迫感、危机感，制定采取更加符合时代要求、更加符合滨州实际的思路措施，努力实现各项工作的新突破。

（一）加快推动转型升级，增强发展内生动力

按照提高经济增长质量和效益的要求，把稳增长与转方式调结构结合起来，把巩固优势与挖掘潜力结合起来，整体提升产业发展水平。一是加快传统产业转型升级。这是转变经济发展方式的主要任务。坚持利用信息技术改造传统产业，提高研发设计、生产过程、生产装备、经营管理信息化水平，加大淘汰落后产能、节能减排力度，鼓励企业兼并重组，争创质量品牌，促进产业链整体升级。二是推动战略性新兴产业健康发展。加强统筹规划，调动各方面的积极性，大力发展新材料、新能源、生物医药、电子信息、高端装备制造等战略性新兴产业，充分发挥在优化产业结构中的带动作用，打造新的经济增长点。三是拓展提升现代服务业。把发展服务业作为优化产业结构的重点，不断提高服务业比重和水平，着力发展创意设计、金融保险、现代物流等生产性服务业，积极培育文化旅游、体育健身、家政服务等生活性服务业。深化企业主辅分离，大力发展面向民生的服务业，不断培育电子商务、网络文化等新兴消费热点。结合老城区改造和新城区建设，创造条件发展总部经济。整合利用国际会展中心、大型场馆等资源，积极承办、兴办大型品牌展会，创新发展会展经济。鼓励支持无棣鲁北文化创意产业园发展。四是大力实施创新驱动。科技创新是提升产业层次的战略支撑，完善以企业为主体、市场为导向、产学研相结合的技术创新体系，重点支持国家和省级企业技术中心、研发中心、重点实验室、院士工作站等科研平台建设。吸引高等院校、科研院所来我市设立分院或研发转化基地。大力推进科技与产业紧密结合，加强技术创新和商业模式创新，不断提高企业自主创新能力。五是支持中小微企业发展。进一步优化发展环境，完善服务体系，继续实施“中小微企业成长工程”，增强生存能力、竞争能力和可持续发展能力。重点支持科技型中小微企业发展，培育壮大一批有实力、成长性好的中小微企业。面对转方式调结构这场硬仗，我们一定要提升境界，主动作为，把精力集中到提高经济增长的质量和效益上来，构建现代产业新体系，激发科学发展动力。

（二）大力发展现代农业，促进农民增收致富

加快发展现代农业，在促进农业增效、农民增收上迈出更大步伐。一是加快实施科技兴农。深化农科对接，力争更多的知名科研机构和重点院校入驻滨州。大力推进“种子工程”，在引进培植知名种业集团、培育优良品种上实现质的突破。创新滨州国家农业科技园区管理机制，加快打造农业新品种、新技术、新装备孵化中心和示范基地。抓好“沃土工程”、“渤海粮仓”项目，开展高产创建，实现科技增粮。加快农业科技创新和技术推广，加强与复旦大学合作，完善“国家863计划——农产品安全追溯系统”，大力推广物联网技术应用。二是加快推进农业对外开放。立足黄三角农业资源特点和优势产业，引进世界最先进的农业技术、生产设备和管理模式，打造现代农业亮点。加强与重点城市和大型农产品流通企业的购销对接，建设农产品预警体系，探索建立高端农产品交易平台，拓宽农产品销售渠道。三是加快培育新型农业体系。创新经营体系，增强农业发展活力。大力培育龙头企业、农民合作组织、新型职业农民等现代农业经营主体，引导支持大型工业企业和社会资本领办农业园区、建设标准化基地。积极稳妥推进土地流转，发展适度规模经营。围绕优势产品，加快培育高效生态农业产业体系。突出抓好沿黄高效生态农业示范带建设，全力打造国家级现代农业示范区。支持现代农业示范园区建设，在去年71家的基础上增加数量，提升标准，打造一批全国领先的亮点园区。四是加快构建现代农业基础支撑。按照旱能浇、涝能排的要求，大搞水利建设，加快推进徒骇河雨洪资源综合利用工程。整合支农资金，统筹抓好农村道路、电网改造、饮水安全等工程。深入实施“林水会战”，改善农村生产生活条件。

加快发展现代农业，有利于保障粮食安全，促进农民增收，有利于推动新农村建设，有利于强化农村基础设施建设、改善公共服务，有利于发展壮大县域经济，实现三次产业协调发展，全力推动由农业大市向农业强市的转变。

（三）实施“黄蓝”两大战略，加快区域联动发展

坚持区域发展与产业培植相结合，优化布局，丰富内涵，放大优势，增强区域综合竞争力。一是加快完善基础设施建设。基础设施是优化产业结构的重要支撑，是经济发展的重要依托。加快滨州港开航运营，尽快融入“环渤海经济圈”，形成“省会城市群经济圈”的出海捷径，进一步提升辐射带动作用。积极推进德大铁路、黄大铁路、寿平铁路、济滨高速以及滨惠、滨阳大道建设，扎实做好滨港铁路二期、济滨城际铁路等前期准备工作，加快各种运输方式的衔接协调和现代物流体系建设。积极推进防潮堤建设，完成秦口河、马颊河和套尔河加固工程。完成占地15.1万亩的未利用地开发项目。全力支持北海经济开发区、无棣、沾化实施沿海开发，大力发展海洋经济，培育海洋优势产业，全面提升基础设施现代化水平，打造形成新的经济增长极。二是推动园区高端提升。推动省级以上开发区主动转型升级，对产业层次低、占地多、贡献小的企业和闲置土地进行清理，提高园区土地集约度，释放发展空间。进一步增强园区承载能力，促进产业集聚与城市功能互补，积极培育一批关联度大、成长性好、带动性强的骨干企业，加快形成一批百亿级、千亿级的产业集群，优化产业结构，提升产业层次，促进产业集群发展。加强能源建设，推动大唐滨州项目顺利开工。三是积极稳妥地推进城镇化。城镇化是现代化的必由之路，是壮大县域经济的重要力量，是富民强市的必然选择。要科学规划，统筹兼顾，做到城乡规划、基础设施、公共服务一体化推进。坚持体制创新，积极探索户籍制度改革，既要土地城镇化、又要人口城镇化，真正使农民转为市民，享受到与市民一样的基本的公共服务。落实扩权强镇政策，培育一批经济强镇、区域重镇、休闲名镇。

机遇千载难逢，必须乘势而上。这是历史的启示，也是时代的召唤。牢牢把握好这次机遇，集中精力谋发展，我们就可以奋发有为，创造更加精彩的奇迹！

（四）推进生态文明建设，凝聚绿色发展优势

加快建设资源节约型、环境友好型社会，必须坚持以人为本、生态优先，实行最严格的资源管理制度，努力形成保护生态环境的产业结构、增长方式和消费模式。一是让“天”更蓝。加快实施“蓝天工程”，抓好节能减排，控制“三废”污染，对敏感区域、化工园区专项整治，让企业主动实施脱硫改造；严管工地，控制扬尘污染；严管秸秆焚烧，确保大气环境质量。二是让“地”更绿。土地是我们的宝贵资源，必须珍惜每一寸国土，严格控制开发强度，加强绿色生态环境建设，抓好万亩生态林场的规划实施，进一步提高森林覆盖率。提升绿色通道标准，建设沾化海韵、博兴麻大湖等湿地公园，给人民群众提供更多的生态休憩场所，给自然留下更多修复空间，给农业留下更多良田。三是让“水”更清。水是一个城市的灵魂，更是生态文明的重要元素。加大雨污管网、污水处理、垃圾焚烧等设施建设，加强对小清河、新立河的综合治理，实施中水回用工程，确保省控河流断面水质稳定达标。做好省级水生态文明城市创建工作。今后，要做到城市道路建设到哪里，雨污管网就配套到哪里。四是让“环境”更美。以创建“国家园林城市”为目标，统筹“地上地下”，精心编制城市环境色彩、水系、景观、风貌等规划。坚持治安、市容、环卫、园林、路灯“五位一体”，建设“智慧城管”。扎实推进生态文明乡村建设和路域综合治理。支持开展国家和省级生态县、生态乡镇创建。积极推进城乡环卫一体化，确保乡镇办全部达标。

推进生态文明建设，目的是提高人民群众的生活质量。资源既属于当代人，也属于后代人。我们的发展既要满足现实需求，更要为子孙后代留下生存空间，实现可持续发展。

（五）深化改革扩大开放，推进体制机制创新

改革是发展的最大动力，必须以更大的勇气和智慧，不失时机地深化重要领域改革，坚决破除一切妨碍科学发展的思想观念和体制机制，构建科学规范、运行有效的制度体系，使制度保障更加坚实、更加有力。一是深化行政体制改革。探索对职责交叉、机构重叠的部门进行整合，加快推进政企分开、政资分开、政事分开、政府与市场中介组织分开。减少行政审批，减少政府对微观经济运行的干预，规范行政行为，深化公共资源交易制度改革，使政府职能切实转变到主要为市场主体服务和创造发展环境上来。加强人大、政协对行政权力的法律监督、民主监督，强化社会监督，重视舆论监督，加强政府内部的专门监督，让权力运行的每一个部位、每一个环节，都置于有效的监督之下。二是深化文化体制改革。推进文化事业单位改革，巩固国有文艺院团改革成果，加大公益性文化投入，调整资源配置，逐步构建公共文化服务体系。落实“十艺节”各项任务。加快文化与科技、旅游、教育融合发展，强化农村文化基础设施建设，丰富农民群众精神文化生活。三是深化医药卫生体制改革。加快推进县级公立医院综合改革，总结经验，扩大试点。做好新农合大病保险工作，加强医保基金收支管理。扩大基本药物制度实施范围。严格医疗市场监管，加快乡村全科医生配备，开展乡村医生全员培训。抓好重点传染病防控。鼓励社会资本举办医疗机构。四是深化财政和投融资体制改革。加强国有资源资产运营管理，探索建立政府投资项目代建制，加强和改进项目管理，实行投资、建设、管理职能分离，提高政府投资效益。深化拓展公共服务购买制，建立政府引导、企业为主、市场运作的供给模式，逐步扩大购买范围。加大股份制银行引进力度，组织好第十二届银企合作促进会。放宽市场准入，优化信用环境，规范融资行为，防范金融风险，严厉打击非法集资和高利贷等不法行为。五是扩大对外开放。不断优化开放结构，提高利用外资水平，吸引外资更多投向高新技术、节能环保、先进制造、新能源、现代农业等领域，大力培育出口品牌，提高出口产品附加值。加快“走出去”步伐，支持企业境外项目合作开发，深化实施自由贸易区合作，以“中日韩地方经济合作示范区”建设为契机，启动滨州中日纺织服装产业园、中韩现代农业园建设，加强与日本川崎、韩国大邱的产业合作。推动引资、引技、引智有机结合，大力引进技术研发人才、经营管理人才来滨州创新创业。

我们一定要放眼全国，融入世界，创新体制机制，扩大合作交流，不断拓展新领域、提升知名度、树立新形象。

（六）切实保障改善民生，加强创新社会管理

时刻把群众冷暖安危放在心上，用心了解社情民意，诚心帮助解决困难，使改革发展成果更多更好地惠及人民群众。一是努力办好人民满意的教育。人民满意的教育必须是高质量的教育。加强教师队伍建设，推进素质教育。加快城区学校建设，合理配置教育资源。加快发展学前教育，均衡发展九年义务教育，提高高中阶段教育质量，创新发展高等教育和现代职业教育。落实教育优先发展地位，促进教育公平，推进教育事业协调发展。二是推动实现高质量的就业。实施更加积极的就业政策，多渠道开发岗位，以创业带动就业。推动“万人就业援助计划”，做好重点群体就业工作，完善就业服务体系，加强职业技能培训，构建和谐的劳动关系，进一步发挥企业在技能人才培养中的主体作用。鼓励高校毕业生面向基层，到生产和服务一线建功立业。三是统筹城乡保障体系建设。完善社保制度体系，做好“三大养老保险”衔接，不断扩大社会保险覆盖面。大力发展慈善事业。完善残疾人社会保障和服务体系。加快发展老年事业，扎实推进养老服务社会化。健全完善医疗保险体系、住房保障体系、社会救助体系，确保“三项基本医保”人口覆盖率稳定在98%以上，确保完成保障性住房建设、农村危房改造任务。四是加强和创新社会管理。建立科学有效的利益协调、诉求表达和权益保障机制。引导更多中介组织参与社会管理。加强警务建设，努力构建和谐社区。全面完成人口与计生工作各项指标。深入开展“扫黄打非”行动。完善防灾减灾、应急管理体系，加强社会治安综合治理、安全生产、食品药品安全工作，维护人民群众的生命财产安全。

今年，扎实做好一些与人民群众密切相关的具体事情：新型农村和城镇居民社会养老保险基础养老金标准，提高到每人每月70元；增加和提高80岁以上老年人生活补贴标准；实现县域“平安校车”全覆盖；新建校舍6.8万平方米，全面完成中小学校舍安全工程；新建改建幼儿园项目90个；资助贫困学生4.6万人；将人均基本公共卫生服务经费标准提高到30元以上；新农合和城镇居民医保补助标准提高到每人每年240元以上；将20类重大疾病纳入大病保险保障范围；完成450个标准化村级卫生场所建设；新增城镇就业3.5万人、农村劳动力转移就业5万人；每个县区新建1处市级大学生创业孵化基地和1处创业园；建设保障性住房4500套，做好廉租住房租赁补贴发放；全市80％以上的行政村配备体育健身设施；完成滨州影剧院改造任务；完成205国道改造示范工程。

各位代表，我们的言行，群众都会看在眼里，记在心上。我们必须尽心竭力，把心思用到发展上来，把精力投入到维护群众利益上来，同心协力，同频共振，奏响和谐发展的最强音，迈出追赶超越的新步伐！

四、加强政府自身建设

各位代表，本着对全市375万人民高度负责的态度，牢记“打铁还需自身硬”的警示，以作风树政风，以政风赢民心，切实加强政府自身建设，努力建设人民满意的政府。

（一）提高素质，依法行政。全面推行政务公开，完善政府决策程序，以新思路、新方式开展工作，处理好政府与市场的关系，既不能管不该管的事情，也不能做该管没管好的事情，既不能“越位”，也不能“缺位”。必须尊重市场规律，依法行政。

（二）转变作风，勤勉理政。深入基层，深入群众，深入困难多、矛盾多的地方，切实帮助基层解决实际问题，把心思用在干事业上，把精力投到抓落实中，力戒形式主义、官僚主义。坚持一切从实际出发，着眼于解决实际问题，以作风转变推动事业发展。

（三）督查问责，从严治政。旗帜鲜明地支持实干者，批评空谈者，惩治腐败者，强化行政监察和审计监督，严肃查处各类违法违纪案件，对欺上瞒下、虚报浮夸、做表面文章的部门和个人，实行最严厉的行政问责，进一步提振政府公信力。

（四）秉公用权，廉洁从政。己不正焉能正人。正确对待和使用好手中的权力，进一步增强公仆意识，积极探索运用政务微博等新媒体，回应社会关切的热点焦点问题，进一步强化群众监督、舆论监督，加强对权力的监督约束，真正做到干部清正、政府清廉、政治清明。

各位代表，生态滨州、美丽滨州、幸福滨州，是全市人民的共同向往，也是全市上下共同的努力方向。让我们紧密团结在以习近平同志为总书记的党中央周围，在市委的坚强领导下，脚踏实地，锐意进取，艰苦奋斗，求真务实，书写无愧于历史和人民的新篇章!
